# Effect of Photobiomodulation on Protein Kinase Cδ, Cytochrome C, and Mitochondria in U87 MG Cells

**DOI:** 10.3390/cells12101441

**Published:** 2023-05-22

**Authors:** Viktória Pevná, Georges Wagnières, Daniel Jancura, Veronika Huntošová

**Affiliations:** 1 Department of Biophysics, Institute of Physics, Faculty of Science, P.J. Safarik University in Kosice, Jesenna 5, 041 54 Kosice, Slovakia; viktoria.pevna@student.upjs.sk (V.P.); daniel.jancura@upjs.sk (D.J.); 2 Laboratory for Functional and Metabolic Imaging, Institute of Physics, Swiss Federal Institute of Technology in Lausanne (EPFL), Station 3, Building PH, 1015 Lausanne, Switzerland; georges.wagnieres@epfl.ch; 3 Center for Interdisciplinary Biosciences, Technology and Innovation Park, P.J. Safarik University in Kosice, Jesenna 5, 041 54 Kosice, Slovakia

**Keywords:** photobiomodulation, mitochondria, protein kinase C, cytochrome c, autophagy, glioblastoma

## Abstract

Photobiomodulation (PBM) therapy is a relatively new modality for the combined treatment of cancer. Pre-treatment of certain types of cancer cells with PBM potentiates the treatment efficacy of photodynamic therapy (PDT). The mechanism of action of this synergetic effect is not yet fully understood. In the present study, we focused on protein kinase Cδ (PKCδ) as a proapoptotic agent that is highly expressed in U87MG cells. The distribution of PKCδ in the cytoplasm was changed and its concentration was increased by PBM using radiation at 808 nm (15 mW/cm^2^, 120 s). This process was accompanied by the organelle specific phosphorylation of PKCδ amino acids (serine/tyrosine). Enhanced phosphorylation of serine 645 in the catalytic domain of PKCδ was found in the cytoplasm, whereas the phosphorylation of tyrosine 311 was mainly localized in the mitochondria. Despite a local increase in the level of oxidative stress, only a small amount of cytochrome c was released from the mitochondria to cytosol. Although a partial inhibition of mitochondrial metabolic activity was induced in PBM-exposed cells, apoptosis was not observed. We hypothesized that PBM-induced photodamage of organelles was neutralized by autophagy maintained in these cells. However, photodynamic therapy may effectively exploit this behaviour to generate apoptosis in cancer treatment, which may increase the treatment efficacy and open up prospects for further applications.

## 1. Introduction

Glioblastoma remains one of the most aggressive cancers. In recent years, several modern approaches to improve conventional therapies aiming to overcome cell resistance have been applied in cancer research. Besides the successes achieved in this field, many side effects are still present in cancer treatment and need to be minimized. For this reason, supportive therapeutic strategies are being developed. One promising approach in this context is based on the use of photobiomodulation (PBM).

PBM uses sub-thermal light irradiances and doses in the red or near infrared region of electromagnetic radiation to stimulate endogenous molecular targets in cells. Although its mechanisms are not yet fully understood, PBM is a very attractive modality, in particular because of the enhancement of metabolic activity, as well as the antiapoptotic and antioxidant responses (stimulation of antioxidant enzymes) it produces [1]. In 1999, Sroka et al. suggested that the protein composition of normal cells differs from that of cancer cells and that the application of PBM to cancer cells causes a decrease in their mitotic rate depending on the applied light dose [2]. Murayama at al. observed an inhibitory effect of PBM on glioblastoma cell proliferation and an influence on cell viability via the mitochondrial respiratory chain [3]. In head and neck squamous cell carcinoma, Ravera et al. found that PBM affects cancer cell homeostasis and the balance between mitochondrial oxidative phosphorylation and glycolysis [4]. This can potentially significantly affect the function of antioxidant enzymes and increase reactive oxygen species (ROS), which, in turn, can lead to apoptosis of cancer cells depending on the dose of light applied. Amaroli et al. isolated mitochondria from bovine liver and observed that a low fluence rate of PBM at 980 nm could uncouple the respiratory chain, leading to higher oxidative stress and the inhibition of adenosine triphosphate (ATP) synthesis, while higher doses could keep the mitochondria coupled and increase ATP production via the activation of mitochondrial complexes III and IV [5].

Oxidative stress increases in numerous pathological conditions due to an imbalance between the antioxidant capacity of cells and the number of free radicals produced. Most ROS are produced in the mitochondria [6,7]. The interaction between molecular oxygen and the respiratory chain has long been proposed and described as a key mechanism leading to the formation of ROS [8]. Depending on the conditions, respiratory chain complexes can produce different types of ROS, mainly in complexes I, II, and III [9], including superoxide and hydrogen peroxide. However, most cells have developed their own protective system, which includes superoxide dismutase (SOD), catalase, and glutathione peroxidase; these are known as antioxidant enzymes [10]. On the other hand, it has been shown that dysfunctional mitochondria can also produce ROS [11]. This may be the case when the level of ROS exceeds the threshold of antioxidant enzyme capacity. Opening of the mitochondrial permeability transition pore results in flooding of ROS into the cytosol, which may lead to more ROS being produced in the nearby mitochondria [12]. This overproduction of ROS can subsequently be detrimental to the entire cell. For this reason, the generation of ROS plays a key role in the treatment of many cancers.

One of the key players in oxidative stress, disruption of mitochondrial respiration, and cell apoptosis, is cytochrome c. The electron transport chain is formed by a series of electron transport proteins located in the inner membrane of the mitochondria (complex I–IV and ATP synthase). These complexes of proteins and molecules transfer electrons from donors to acceptors, creating a concentration gradient across the inner mitochondrial membrane. Cytochrome c presents a unique protein that is not part of the large complex, but diffuses freely across the inner mitochondrial membrane and transports electrons between complex III and complex IV (cytochrome c oxidase) of the respiratory chain [13]. However, cytochrome c plays also very important role in the initiation of apoptosis. The mechanism of release of this protein from the mitochondria is well studied in the context of cell death and proliferation, and has been described in the review by Garrido et al. [14]. In order to induce apoptosis in cells through the release of cytochrome c, the outer membrane of the mitochondria must be permeabilized. However, a nonapoptotic (cell survival) model has been proposed in which small amounts of cytochrome c are released from the mitochondria by a specific mechanism without permeabilization of the mitochondrial outer membrane and other apoptotic factors [14]. The mitochondria have been shown to undergo morphological changes related to mitochondrial division, fission mechanism, and activation of proapoptotic proteins before cytochrome c is released from the mitochondria into affected cells [15,16]. Ott et al. proposed a two-step process of cytochrome c release from the mitochondria [17]. First, cytochrome c detaches from the inner mitochondrial membrane, and then the outer mitochondrial membrane is permeabilized, in which proapoptotic proteins of the BCL-2 family play an important role. Permeability transition pores in the outer mitochondrial membrane are formed with Bax (Bcl-2-associated X protein) and Bak (Bcl-2 homologous antagonist/killer) proteins. These pores can be then activated with BID (BH3 interacting domain death agonist) protein to initiate permeabilization of the outer mitochondrial membrane [18].

It has also been reported that oxidative stress signals regulate cell proliferation and death [19]. We recently demonstrated the important role of ROS-induced protein kinase C (PKC) signaling when applying PKC regulators and light to cancer cells [20,21,22]. Phorbol 12-myristate 13-acetate (PMA) has long been known as a tumor promoter that interacts with PKC and regulates its activity [23]. Later, the inhibitory effect of phorbol ester on cancer cells and its apoptosis induction were observed [24,25,26]. PMA caused degradation of the mitochondrial membrane potential through the inhibition of complex I as well as pyruvate dehydrogenase, as well as the induction of ROS [27]. Moreover, PKCα and PKCδ have been reported to be translocated from the cytosol to the plasma membrane and mitochondria of the cell under this effect [27]. Although few studies have investigated the effect of PBM on Ca^2+^ release [28,29], it has been shown that PMA also generates superoxide anions and impairs the buffering of cytosolic Ca^2+^. This may enhance the PKC-dependent activation of phagocytic NADPH oxidase [30]. PMA may also play a role in the mitochondrial opening of ATP-dependent K^+^ channels [31]. Majumer et al. reported that phorbol ester induces the translocation of PKCδ from the cytoplasm to the mitochondria, and that this translocation in turn causes the release of cytochrome c, activation of caspase 3, and induction of apoptosis [32].

In the present work, we aimed to demonstrate the effect of PBM at 808 nm on PKCδ and its phosphorylated forms in isolated fractions of U87MG cells. Morphological changes in mitochondrial organization were observed through the fluorescence of vital and immunostained mitochondria. Finally, mitochondrial clouds associated with cytochrome c release were studied in the perinuclear region of these cells after the application of PBM and PMA.

## 2. Materials and Methods

### 2.1. Cell Cultures and Therapeutical Protocol

Human glioma cells U87 MG (Cells Lines Services, Eppelheim, Germany) were grown in the dark under a humidified atmosphere of 5% CO_2_ at 37 °C to 80% confluency in Dulbecco’s Modified Eagle Medium (DMEM, high glucose, GlutaMAX^TM^, with pyruvate, Gibco-Invitrogen, Life Technologies Ltd., Paisley, UK) supplemented with 10% fetal bovine serum (FBS, Gibco-Invitrogen, Life Technologies Ltd., Paisley, UK) and 1% *(w/w*) penicillin/streptomycin (Gibco-Invitrogen, Life Technologies Ltd., Paisley, UK).

Several protocols were tested:

(1) Cells were treated with 10 µM rotenone (Sigma-Aldrich, St. Louis, MO, USA) for 24 h. Furthermore, these cells were irradiated with PBM at 808 nm and 1.8 J/cm^2^ (120 s with an irradiance of 15 mW/cm^2^). The effect of PBM was evaluated immediately (0 h) after irradiation and 1–5 h after irradiation.
(2) The cells were treated with PBM at 808 nm and 1.8 J/cm^2^, and subsequently 1 µM PMA (Sigma-Aldrich, St. Louis, MO, USA) was administered to the cells for 5 h.
(3) The cells were treated with PBM at 808 nm and 1.8 J/cm^2^. The effect of PBM was evaluated immediately (0 h) after irradiation and 1–5 h after irradiation.

The final amount of DMSO applied to the cell culture medium was less than 0.1%.

The light emitted by an 808 nm infrared diode laser (MDL-III-808/1~2500 mW, Changchun New Industries Optoelectronics Tech. Co., Ltd., Changchun, China) was coupled to a frontal light diffuser (FD1, Medlight SA, Ecublens, Switzerland) to generate homogeneous illumination spots.

The cells were seeded in Petri dishes (3.5 cm in diameter) embedded with coverslips for confocal microscopy and in 25 cm^2^ flasks. Laser light covered the surface of the irradiated dish and flask. PBM treatment was performed from the bottom.

Cell viability (related to the mitochondrial metabolic activity of formazan production) was determined through the MTT assay according to a previously published protocol [33] in samples with different PBM irradiation times (0–600 s), and the detection time of the cell response after PBM ranged from 0–24 h.

### 2.2. Vital- and Immuno-Fluorescence Confocal Microscopy

Fluorescence images were acquired using a confocal fluorescence microscope system (LSM 700, Zeiss, Oberkochen, Germany) equipped with a 40X water immersion objective (NA 1.2, Zeiss) and a CCD camera (AxioCam HRm, Zeiss). Fluorescence images were analyzed using Zen 2011 software (Zeiss). Samples were excited with lasers at 405, 488, and 555 nm.

Vital staining: The mitochondria were stained with 5 µM Rhodamine 123 (Rh123, Sigma-Aldrich, Darmstadt, Germany) for 15 min (excitation at 488 nm and emission in a spectral range of 490–530 nm). The cell nuclei were stained with 10 µg/mL Hoechst 33342 (ThermoFisher Scientific, Waltham, MA, USA) for 30 min (excitation at 405 nm and emission in a spectral range of 450 ± 40 nm). The plasma membrane of the cells was stained with 2.5 μg/mL CellMask^TM^ Orange (ThermoFisher Scientific, Waltham, MA, USA) for 5 min (excitation at 555 nm and emission in the spectral range > 580 nm). Lipid peroxidation was visualized with a lipid peroxidation assay kit (ab243377, Abcam, Cambridge, UK) according to supplier protocol. MitoTracker Orange CMTMRos (1 µM, 15 min, ThermoFisher Scientific, Waltham, MA, USA) was used to label the mitochondria and mitochondrial stress in the cells. The fluorescent dyes were rinsed off the cells before microscopy.

Immunostaining: The cells were fixed after treatment with ice-cold (−20 °C) acetone (Centralchem, Bratislava, Slovakia) for 5 min at −20 °C. The cells were washed in ice-cold phosphate-buffered saline (PBS, Sigma-Aldrich, Darmstadt, Germany) and blocked in 5% bovine serum albumin (BSA, Sigma-Aldrich, Darmstadt, Germany) in PBS at room temperature (25 °C) for 1 h. The cells were then incubated with primary antibodies dissolved in 5% BSA with anti-Giantin (1:300, ab80864, Abcam, Cambridge, UK), anti-cytochrome c (1:300, ab110417, Abcam, Cambridge, UK), anti-PKCδ (1:300, ab182126, Abcam, Cambridge, UK), and anti-LC3B (1:300, ab221794, Abcam, Cambridge, UK) for 1 h at room temperature. After incubation, the cells were washed with ice-cold PBS. Then, the secondary antibody conjugated with AlexaFluor 488 (1:1000, ab150077, Abcam, Cambridge, UK), AlexaFluor 546 (1:1000, A-11030, Thermo Fisher Scientific, Waltham, MA, USA), and AlexaFluor 405 (1:1000, ab175652, Abcam, Cambridge, UK), and diluted in 1% BSA, was applied to the cells for 1 h at room temperature. Embedding medium containing 4′,6-diamidino-2-phenylindole (DAPI, ab104139, Abcam, Cambridge, UK) was applied to stabilize the fluorescence signal and counterstain the nuclei. Fluorescence was detected as follows: AlexaFluor 488 (excitation at 488 nm and emission in the spectral range 490–530 nm), AlexaFluor 546 (excitation at 555 nm and emission in the spectral range >580 nm), AlexaFluor 405, and DAPI (excitation at 405 nm and emission in the spectral range 450 ± 40 nm and >580 nm for DAPI).

### 2.3. Western Blot Analysis

Two protocols were performed to obtain lysates of different cell fractions and a total cell lysate.

(1) Lysates from different cell fractions: 10^6^ cells were kept on ice for 15 min in a lysis buffer containing an inhibitor cocktail (2 × 1:100, Halt™ Protease and Phosphatase Inhibitor Cocktail, ThermoFisher Scientific, Waltham, MA, USA). The lysis buffer consisted of 20 mM 4-(2-hydroxyethyl)piperazine-1-ethanesulfonic acid, 10 mM KCl, 2 mM MgCl_2_, 1 mM ethylenediamine-tetraacetic acid, and 1 mM ethylene glycol-bis(2-aminoethyl ether)-N,N,N′,N′-tetraacetic acid (all of the chemicals were purchased from Sigma-Aldrich, St. Louis, MO, USA). Three fractions were prepared. The cells were homogenized with a syringe needle and kept on ice for 20 min. The cell homogenates were collected and centrifuged at 3000 rpm for 5 min at 4 °C to obtain two fractions: (A) supernatant containing cytosol and mitochondria, and (B) pellet containing membranes and nuclei. These two fractions were further separated from excess unwanted cell compartments. (A) The supernatant was centrifuged for 5 min at 8000 rpm and 4 °C. The cytosolic fraction was recovered as the supernatant. The mitochondria were collected in the pellet, which was then sonicated on ice for 30 s in 0.1% sodium dodecyl sulfate (Sigma-Aldrich, St. Louis, MO, USA) and 50 mM Tris-(hydroxymethyl) aminomethane hydrochloride (Tris, VWR, Leuven, Belgium) buffer to obtain the mitochondrial fraction. (B) The pellet was resuspended in the lysis buffer and centrifuged at 3000 rpm for 10 min at 4 °C. The nuclear and membrane fraction, designated f_3_, was obtained through sonication of the pellet in 0.1% sodium dodecyl sulfate and Tris.

(2) The total lysate of the cells: 10^6^ cells were lysed and homogenized in a radioimmunoprecipitation buffer (RIPA) (150 mM sodium chloride, 1% Triton X-100, 0.5% sodium deoxycholate, 0.1% sodium dodecyl sulfate, 50 mM Tris, pH 8; all of the chemicals were purchased from Sigma-Aldrich, Darmstadt, Germany) with an inhibitor cocktail (2 × 1:100, Halt™ Protease and Phosphatase Inhibitor Cocktail, ThermoFisher Scientific, Waltham, MA, USA). The total lysates (120 μg/mL of total protein amount) were diluted to 60 μg/mL of final protein amount in 2× Laemmli buffer (Sigma-Aldrich, Darmstadt, Germany); 10 μL (per lane) from this solution was loaded onto 10% or 12% polyacrylamide gels and subjected to electrophoresis. The proteins were transferred to a nitrocellulose membrane (0.22 μm; AppliChem; Darmstadt, Germany). Immunodetection was performed using a Western Breeze Chromogenic Kit (ThermoFisher Scientific, Waltham, MA, USA). The proteins in the membrane were blocked with 5% BSA for 1 h at room temperature, washed, and then incubated overnight at 4 °C with primary antibodies of anti-LC3B (1:3000, ab221794, Abcam, Cambridge, UK), anti-PKCδ (1:300, ab182126, Abcam, Cambridge, UK), cytochrome c apoptosis WB antibody cocktail (1:1000, ab110415, Abcam, Cambridge, UK), anti-STAT3 antibody (1:1000, ab109085, Abcam, Cambridge, UK), autophagy analysis (ATG16L1, SQSTM1, LC3B, Ubiquitin, MGPR), and an antibody sampler panel (1:1000, ab269811, Abcam, Cambridge, UK). Anti-GAPDH (1:1000, ab181602, Abcam, Cambridge, UK) and anti-Lamin A/C (4C11) (mAb #4777, Cell Signaling Technology, Danvers, MA, USA) were determined as the house-keeping proteins. For protein visualization, the secondary antibodies from the Western Breeze Chromogenic Kit were used to detect the rabbit and mouse primary antibodies. Analysis of the optical densities (O.D.) of the secondary antibodies on the membrane was performed using ImageJ software (1.48v) [34]. O.D. values were normalized to the total protein content, as visualized with the Coomassie^®^ Brilliant Blue R 250 (SERVA Electrophoresis, Heidelberg, Germany) stain solution in gel.

### 2.4. Fluorescence Lifetime Imaging of Mitochondrial Oxidative Stress Level

Fluorescence lifetime images of the cells labeled with MitoTracker Orange CMTMRos were detected using a dedicated microscopy system. An inverted fluorescence microscope (Zeiss AxioObserver Z1, Zeiss, Oberkochen, Germany) was equipped with a 40× water immersion objective (NA = 1.2, with adjustable coverslip correction) and connected to a fluorescence lifetime detection module (FLIM, DSC-120 Dual Channel Confocal Scanning system, Becker and Hickl GmbH, Berlin, Germany). The samples were excited with a pulsed NKT-Super-K Extreme laser (40 MHz, 5ps pulse, NKT Photonics, Birkerod, Denmark) at 555 nm (2% of total power). Fluorescence lifetimes were detected with a HPM-100-50 hybrid detector (Becker and Hickl GmbH, Berlin, Germany) in a 25 ns time range. The emission was filtered with LP 488 and BP 624 ± 20 nm (NT67–035, EDMUND Optics, Barrington, NJ, USA). Analysis of the lifetimes was done with SPC image analysis software (Becker and Hickl GmbH, Berlin, Germany). The quality of the fits was graphically checked by plotting the residuals and χ^2^~1.

### 2.5. Statistical Analysis

The experiments were repeated at least twice. The level of significant difference was determined using Student *t*-test: * *p* < 0.05, ** *p* < 0.01, and *** *p* < 0.001.

## 3. Results and Discussion

**Figure 1:**
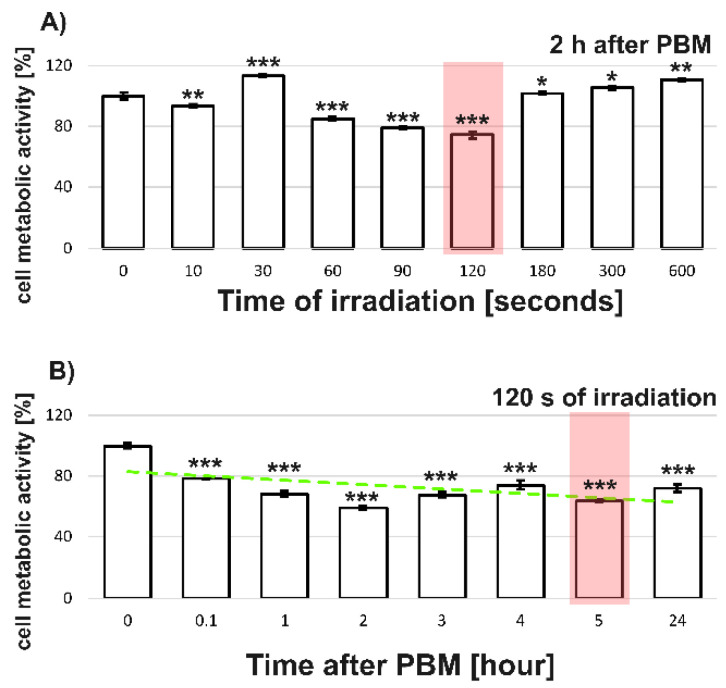
MTT assays of U87MG cells irradiated with 808 nm: (**A**) different irradiation durations when the response is assessed 2 h after PBM, (**B**) different times after PBM when the cells are illuminated for 120 s. The green slashed line is presented for visual support and represents the linear fit of trend. Red rectangles identify selected parameters for further studies. The level of significant difference was determined using Student *t*-test: * *p* < 0.05, ** *p* < 0.01, and *** *p* < 0.001.

We examined the response of U87MG cells to PBM after illumination with radiation at 808 nm at different light doses. The MTT assay performed relatively shortly (2 h) after PBM mainly reflected the metabolic activity of the mitochondria in the cells (Figure 1A). Significant differences in metabolic activity were observed between the treated- and non-treated-PBM cells (0 s in Figure 1A). Overall, a decrease in metabolic activity of about 80% was observed after a 120 s irradiation. Longer irradiations (180–600 s) resulted in a significant increase in metabolic activity of the U87MG cells. There is evidence in previous works by other groups that PBM can inhibit the proliferation of cancer cells in certain conditions [2,3]. For this reason, and our interest in identifying the possible sources of the inhibitory effect of PBM on the metabolic activity of U87MG cells, we chose an irradiation time of 120 s, corresponding to a light dose of 1.8 J/cm^2^ (highlighted in red in Figure 1A).

Next, we applied selected PBM protocols (Section 2.1) to U87MG cells and examined the metabolic activity of the cells using an MTT assay at various time points ranging between 0.1 to 24 h after PBM (Figure 1B). As expected, we found a significant decrease in the metabolic activity of the cells in all of the cases examined compared with the activity of the non-treated cells. Based on these results, we chose an appropriate detection window (~5 h) that was correlated with inhibition of the metabolic activity of about 70% compared with the nonirradiated control (shown as 0 h). There is a slight discrepancy in the MTT tests in Figure 1A,B. This is because these protocols were performed in separate experiments. We would like to point out here that the MTT test may be partially influenced by hydrogen peroxide and the enzymes active in the defense against oxidative stress by PBM. 

### 3.1. Level of Protein Kinase Cδ and Autophagy in U87MG Cells after PBM

**Figure 2:**
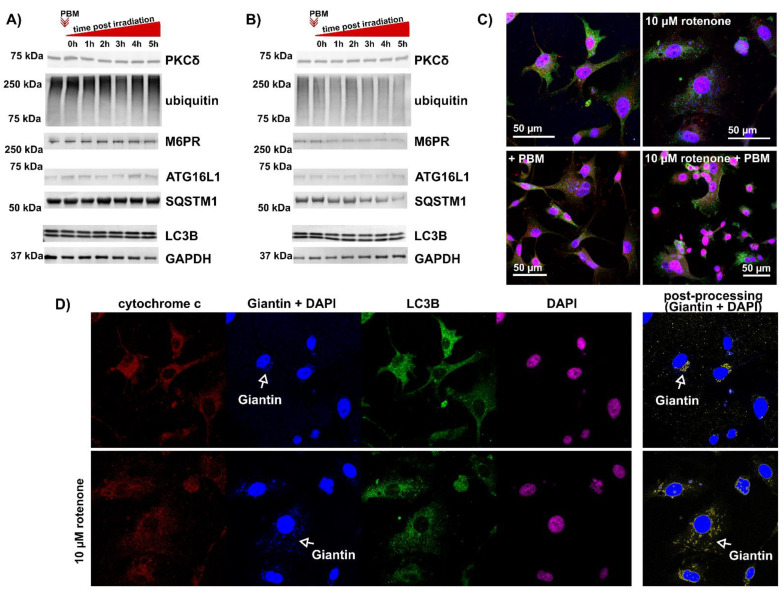
Evidence of autophagy observed in U87MG cells treated with PBM. (**A**) Total protein lysate is collected immediately or 1–5 h after PBM. (**B**) Cells exposed to rotenone for 24 h and then treated with PBM. The total protein lysate is collected immediately or 1–5 h after PBM. (**C**) Representative fluorescence images of cells exposed to PBM and rotenone and immunolabeled with antibodies against cytochrome C (red), giantin (blue), and LC3B (green). Cell nuclei are labeled with DAPI (blue and pink). The representation of each channel is shown in (**D**). White arrows mark the position of the Golgi apparatus. Giantin and DAPI images are subjected to postprocessing (subtraction of DAPI image from Giantin + DAPI image) to highlight the Golgi apparatus in yellow.

PKCδ and the autophagic markers LC3B, ATG16L1 (related to autophagosome formation), SQSTM1 (related to autophagosome degradation), M6PR (related to lysosomes), and ubiquitin were examined in the total lysates of U87MG cells exposed to PBM. No significant effects on the levels of these proteins were detected compared with their levels in nonirradiated sample (see Figure 2A). As many groups reported a beneficial effect of PBM on damaged mitochondria [35,36], we exposed U87MG cells to 10 µM rotenone for 24 h to induce an acute toxic effect and the inhibition of complex I of the respiratory chain [37,38]. Apart from the remarkably low levels of the tested proteins, no significant effect of PBM on the global level (in total cell lysates) of PKCδ and autophagic proteins was detected (see Figure 2B). The results of the Western blot analysis were confirmed by visualization of LC3B by fluorescent immunostaining, as presented in Figure 2C. LC3B foci, as green spots, were observed in all of the cases examined. However, a difference was observed in the distribution of the mitochondria and Golgi apparatus (Figure 2C,D) visualized with cytochrome c (in red colour) and Giantin (in blue colour). Mitochondrial fission and fragmentation of the Golgi apparatus were observed in the cells exposed to rotenone (Figure 2D). After PBM, more red (corresponding to cytochrome c) and diffuse pixels were observed in the perinuclear region. Therefore, we first examined the mitochondrial morphology and oxidative stress generated in these organelles and then focused on the distribution of cytochrome c and PKCδ in the cells.

### 3.2. Morphology of the Mitochondria and Level of Oxidative Stress in the Mitochondria of U87MG Cells after PBM

**Figure 3:**
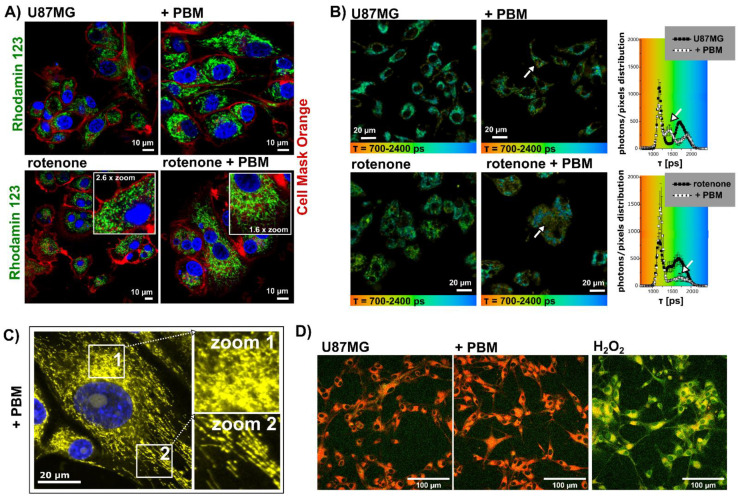
Representative fluorescence images of U87MG cells treated with PBM, rotenone, and rotenone+PBM, (**A**) labeled with Rhodamine123 (green, mitochondria), Cell Mask Orange (red, plasma membrane), and Hoechst (blue, cell nuclei), and (**B**) FLIM images of MitoTracker Orange CMTMRos and distribution histograms of its fluorescence lifetime in the cells. The arrows point to changes due to PBM. (**C**) Distribution of the mitochondria in cells treated with PBM (MitoTracker Orange CMTMRos in yellow, cell nuclei are marked with Hoechst in blue). Zoom 1 shows mitochondrial clouds and oxidative stress characterized by the diffuse distribution of the fluorescent probe. Zoom 2 shows fused long fibrillar mitochondria. (**D**) Representative images of cells exposed to PBM and H_2_O_2_ labeled with a fluorescent probe (ab243377) sensitive to lipid peroxidation, shown by a color change from red to green.

The mitochondria in the U87MG cells were labelled with Rhodamine123, the plasma membrane was labelled with Cell Mask Orange, and the nuclei were stained with Hoechst to better identify individual cell compartments. Tubular structures of the mitochondria were found in the cells without PBM treatment. After irradiation, the mitochondria began to form clouds by fusing individual tubes together (Figure 3A). In the cells exposed to rotenone, the mitochondria underwent fission and spherical patches were observed. The application of PBM to rotenone-treated cells resulted in significant fusion of the mitochondria, as shown by the existence of a mitochondrial network (Figure 3A). We did not observe any impairment of the mitochondrial membrane potential (related to Rhodamine123 fluorescence intensity decreasing) through the application of rotenone or PBM.

The fluorescence lifetime of certain molecular probes is a sensitive parameter that can be used to characterize the local environment and is independent of the concentration of the fluorescent molecule [39]. We have developed a method to visualize the extent of oxidative stress in living cells by measuring the fluorescence lifetime of the MitoTracker Orange CMTMRos [21]. In this approach, the fluorescence lifetime of this mitochondrial probe increased with the extent of oxidative stress. Figure 3B shows the fluorescence lifetime imaging (FLIM) of U87MG cells exposed to rotenone and PBM. Two main populations of fluorescence lifetime distribution (histograms on the right side of Figure 3B) can be observed in the FLIM images—longer lifetimes range between 1500–2000 ps and short lifetimes range between 1000–1400 ps. While the shorter lifetimes persisted after PBM, the longer lifetimes partially disappeared. Localization of the molecules with longer lifetimes, represented by images with a green-blue color, can be found in the perinuclear regions. Molecules with short lifetimes are mainly distributed in the mitochondria, in the cytoplasm, and close to the plasma membrane. Variations in fluorescence lifetimes are indicated by white arrows. 

To better identify the state of the mitochondria after PBM (without light perturbations induced by the detection system), the cells exposed to PBM were labelled with MitoTracker Orange CMTMRos and fixed at −20 °C in a cold methanolic solution. In these cells, two areas can be identified: (1) mitochondrial clouds in the perinuclear region where the fluorescence is diffuse and where partial fission of the mitochondria is observed (zoom 1 in Figure 3C), and (2) long fused tubular mitochondria distant from the nucleus distributed in the cytosol (zoom 2 in Figure 3C). In view of these results, a dual effect of PBM is suggested. The increase in oxidative stress occurs preferentially in the perinuclear region, and lipid peroxidation is not observed in the cells after PBM (see Figure 3D). We hypothesized that antioxidant enzymes in cells such as catalase, superoxide dismutase, and thioredoxin rapidly become active in the first line of defense mechanisms.

### 3.3. Cytochrome c and PKCδ Distribution in U87MG Cells after PBM

**Figure 4:**
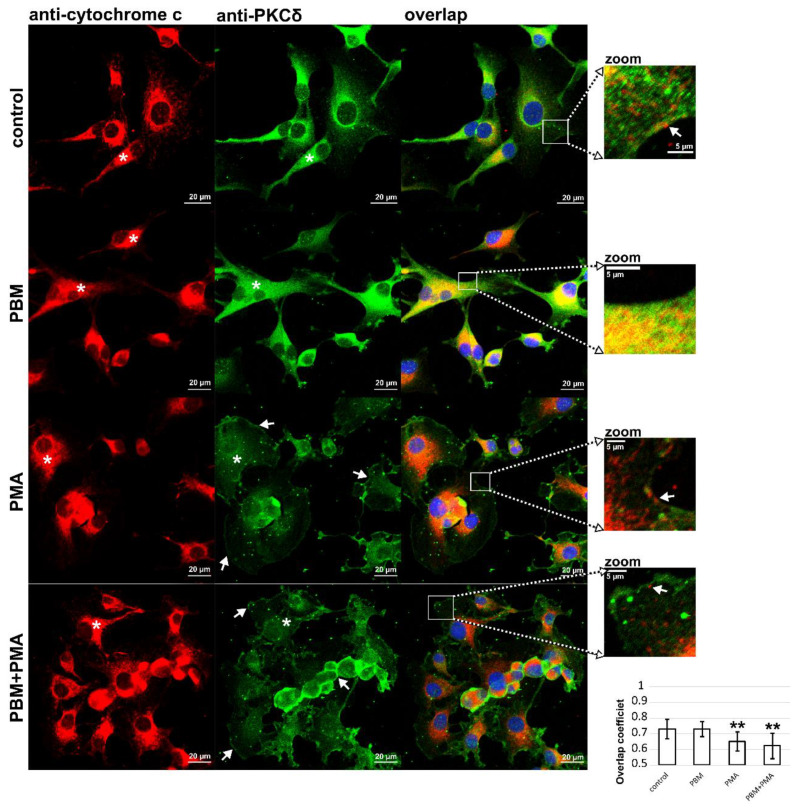
Representative fluorescence images of U87MG cells treated with PBM, PMA, and PBM+PMA. Cells are immunolabeled with antibodies against cytochrome C (red) and PKCδ (green). Cell nuclei are stained with Hoechst (blue). White asterisks indicate perinuclear areas where the mitochondria and endocytotic vesicles are localized. Zoomed areas show the localization of cytochrome c and PKCδ near the plasma membrane, denoted by white arrows. Histograms show overlap coefficients determined on the U87MG cells. Student’s *t*-test is used to determine significant differences: ** *p* < 0.01.

Cytochrome c is a sensitive marker whose release from the mitochondria predicts the rate of apoptosis induction in cells. The distribution of cytochrome c in U87MG cells exposed to PBM is shown in Figure 4. Cytochrome c is dominantly localized in the mitochondria of U87MG cells. However, crowded areas exist near the nuclei, indicated by the white asterisks in Figure 4. After PBM, the distribution of cytochrome c is more diffuse and fills the cytosol. Furthermore, these areas appear fuzzier, which may be related to the local release of cytochrome c. In addition to PBM, the effect of PMA on U87MG cells was also examined. In cells exposed to PMA, the distribution of cytochrome c was comparable to that after PBM treatment. The application of PBM prior to PMA exposure did not significantly alter this distribution.

Strong effects of PBM on the mitochondria in different cell types have been reported [40]. In the present work, we focused on glioblastoma cells U87MG, which are known to express two PKC isoforms whose interplay determines cell death. In our previous studies, we showed that light combined with photosensitizers can positively or negatively affect the activity of two PKC isoforms [22,41,42]. PKCα is a serine–threonine kinase whose activation leads to its relocalization from the cytoplasm to the plasma membrane [41]. It has been shown that PKCα enhances the activity of proapoptotic proteins and inhibits the antiapoptotic ones [43]. This behavior has been observed in response to PMA, similar to its antipode PKCδ [22,41]. However, PKCδ was observed in the mitochondria of cells exposed to phorbol esters [32]. This localization may be responsible for maintaining mitochondrial oxidative stress at a critical level before apoptosis is triggered. In our recent study, we observed that the irradiation of glioblastoma cells with an 808 nm laser light resulted in significant enhancement of hypericin-induced photodynamic therapy applied several hours after PBM [33]. In the present study, we focused on the shorter period (after PBM before induction of PDT).

PKCδ was distributed in the cytoplasm of U87MG cells unaffected by irradiation (Figure 4). PBM using irradiation at 808 nm resulted in an increase in PKCδ expression with a non-specific distribution that was found throughout the cell. The overlap coefficient determined from the colocalization analysis reached similar values of 0.7 without and with PBM. Colocalization of the mitochondria (cytochrome c) and PKCδ is shown in the zoomed images. Overlaps can be seen by the yellow color of the pixels.

In contrast with PBM, cells exposed to PMA showed a weak green fluorescence intensity (related to PKCδ) in the cytosol and a strong one in the plasma membranes, as shown with the white arrows in Figure 4. The overlap coefficients of cytochrome c and PKCδ for the cells exposed to PMA decreased significantly (see histograms in Figure 4). This observation suggests that only a small fraction of PKCδ and cytochrome c are located at the same position on the images. These areas are located in the perinuclear regions, but some mitochondria are localized near the plasma membrane, as shown in the zoomed images.

Fluorescence microscopy showed that the expression of proapoptotic PKCδ increased after PBM in the U87MG cells. Its homogeneous and broad distribution in cells increases the probability that it is also involved in mitochondrial processes and oxidative phosphorylation. 

During the initiation of apoptosis, PKCδ is proteolytically activated by caspase 3 [44]. PKCδ is cleaved into two fragments that are constitutively active and initiate apoptosis [45]. These fragments are responsible for the disassembly of the nuclear lamina during apoptosis [46]. PKCδ is one of the proteins activated by lipids, and it is equally probable that not only the mitochondria, but also the endoplasmic reticulum, may be a source of oxidative stress that increases PKCδ activation. This is consistent with the observations of Larroque-Cardoso, who showed that PKCδ is involved in apoptosis induced by oxidative stress generated in the endoplasmic reticulum by the oxidation of lipoproteins [47].

Reactive oxygen species have been implicated not only in the activation of PKCδ, but also in its phosphorylation, which is specifically attributed to tyrosine 311 (Tyr311) [48,49]. Phosphorylation of this site (Tyr311) has been reported to play an important role in platelet function and vascular smooth muscle cell hypertrophy [50,51]. Recently, PBM was applied to smooth muscle cells and implicated in improved prognosis in abdominal aortic aneurysms and blood flow in ischemic tissue [52,53].

### 3.4. Phosphorylation of PKCδ in U87MG Cells after PBM

Fluorescence microscopy (Figure 4) revealed an increase in PKCδ protein levels in the cells after PBM. However, a change in PKCδ content in the total cell lysate in response to PBM was not observed (Figure 2). This motivated us to isolate organelle and cytosolic fractions and to determine PKCδ and its phosphorylation forms from these fraction lysates. We identified three fractions in the samples: cytosolic, mitochondrial, and f_3_ (containing plasma membrane and nuclear proteins).

**Figure 5:**
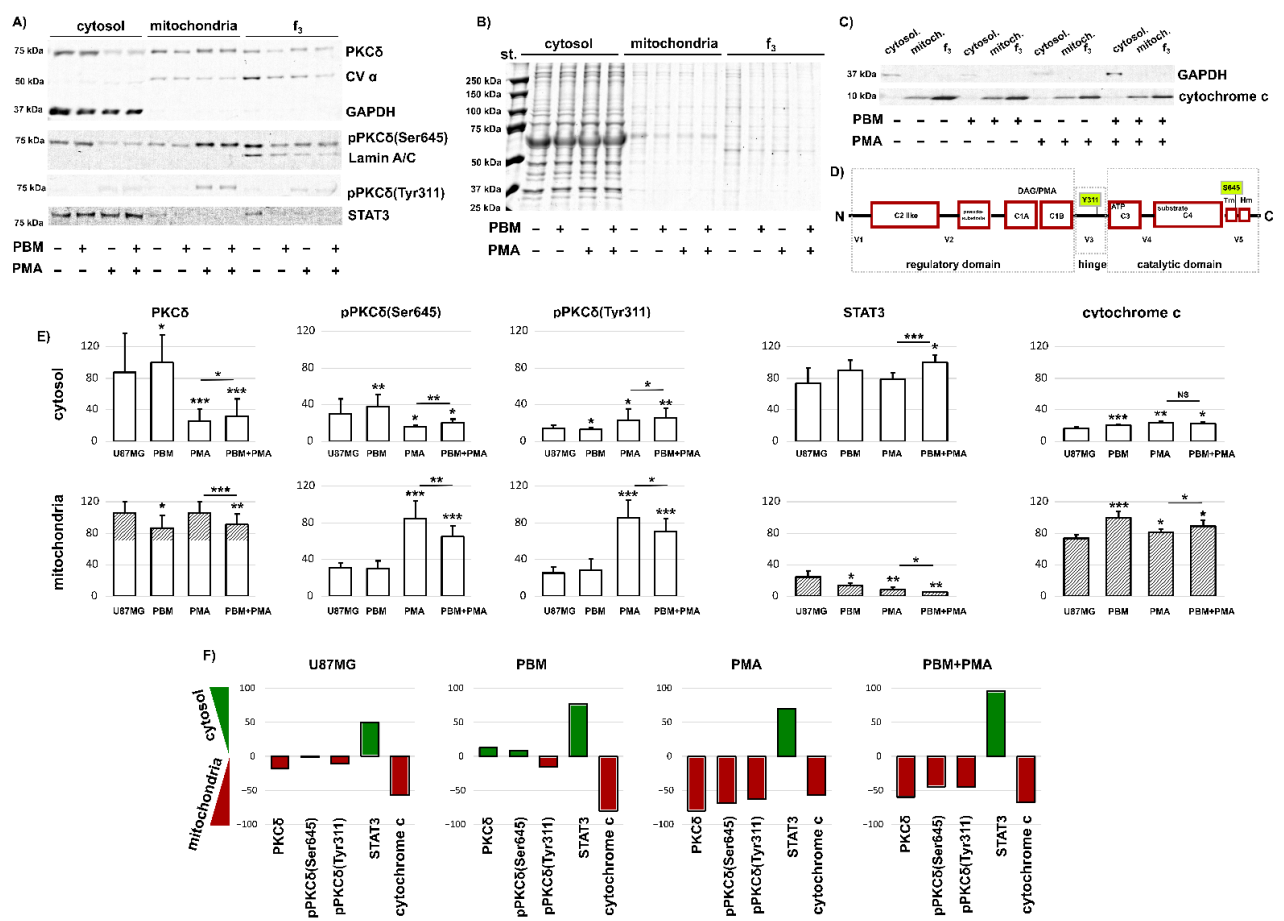
Protein levels of (**A**) PKCδ, pPKCδ(Ser645), pPKCδ(Tyr311), STAT3, CVα, GAPDH, and Lamin A/C. (**B**) Total proteins. (**C**) GAPDH and cytochrome c in three fractions: cytosolic, mitochondrial, and f_3_ from U87MG cells treated with PBM and PMA, as indicated. (**D**) Schematic representation of PKCδ and localization of the PMA binding site and amino acids Tyr311 and Ser645. (**E**) Analysis of the detected proteins: O.D. is normalized to the total protein levels. (**F**) Difference in protein levels in cytosolic and mitochondrial fractions (cytosol−mitochondria). Green histograms represent positive and red histograms represent negative values corresponding to a stronger localization of proteins in the cytosol and mitochondria, respectively. The level of significant difference was determined using Student *T*-test: NS-not significant, * *p* < 0.05, ** *p* < 0.01, and *** *p* < 0.001.

Figure 5A shows all three fractions isolated from U87MG cells exposed to PBM, PMA (which serves as a positive control here), and both PBM and PMA. GAPDH (glyceraldehyde-3-phosphate dehydrogenase) was identified only in the cytosolic fraction, CVα was observed in the mitochondrial and f_3_ fractions, whereas Lamin A/C was found only in the f_3_ fraction. On this basis, we were able to correctly identify the cytosolic and mitochondrial fractions whose proteins were further analyzed. 

Significant amounts of PKCδ were found in the cytosol and mitochondria of U87MG cells without and with PBM exposure (Figure 5A). However, in the cells exposed to PMA, high amounts of PKCδ were found in the mitochondria, and significantly less were found in the cytosol. A very similar observation was made for pPKCδ(Ser645) (Figure 5A,E). In contrast with these observations, pPKCδ(Tyr311) was observed mainly in the mitochondria and to a lesser extent in the cytosol (Figure 5A,E). The localization of the phosphorylation site on PKCδ is shown in Figure 5D. Tyr311 is localized in the hinge region connecting the regulatory domain to the catalytic domain. Its phosphorylation leads to conformational changes so as to allow caspase 3 to access the PKCδ cleavage site [54]. On the other hand, Ser645 is the conserved phosphorylation site of the turn motif in the catalytic domain of PKCδ, and its phosphorylation is required for catalytic activation [55].

There was not much difference between the studied protein levels in the irradiated and nonirradiated cells. However, PBM stimulated PKCδ and pPKCδ(Ser645) in the cytosol and reduced PKCδ, pPKCδ(Ser645), and pPKCδ(Tyr311) in the mitochondria of the cells exposed to PMA (Figure 5E). This effect is better illustrated by the difference between the cytosolic and mitochondrial fractions in Figure 5F. Whereas the phosphorylation of Ser645 was more abundant in the cytosol, the phosphorylation of Tyr311 was more abundant in the mitochondria after PBM. In cells exposed to PMA and PBM+PMA, PKCδ, pPKCδ(Ser645), and pPKCδ(Tyr311) were mainly located in the mitochondria (Figure 5F).

The signal transducer and activator of transcription 3 (STAT3) is highly expressed in glioblastoma cells and often contributes to tumorigenesis by inhibiting apoptosis [56]. Here, we observed enormous STAT3 levels in the cytosol in all of the samples treated by PBM (Figure 5A). While weak STAT3 levels were detected in the mitochondria of untreated cells and cells after being treated with PBM, exposure to PMA resulted in the disappearance of STAT3 from the mitochondria (Figure 5E,F). Shen et al. reported that STAT3, localized in the cytoplasm, plays a role in suppressing autophagy by interacting with protein kinase R [57]. We observed autophagy and LC3B-containing vesicles in U87MG cells without and with PBM (Figure 2). This may suggest that STAT3 is increased in the cytoplasm due to an antiapoptotic response, autophagy, or the stimulation of cell growth by PBM.

Mitochondrial cytochrome c was detected in all of the cases examined (Figure 5C). However, the application of PBM to U87MG cells increased mitochondrial cytochrome c levels (Figure 5E,F). Cytosolic cytochrome c was detected in PBM-treated cells and in cells exposed to PMA. However, the level of this cytochrome c was very low. This low amount can be attributed to the fraction we observed in the perinuclear region through confocal fluorescence microscopy. The amount of cytochrome c released from the mitochondria, although induced by the local production of oxidative stress, was not sufficient to induce global apoptosis.

The observations we made after PBM in glioblastoma cells show that this treatment did not result in dramatic changes in the cells. However, local damage was induced, and a change in oxidative stress was generated (see FLIM results) in the mitochondria, likely leading to the inhibition of the mitochondrial metabolic activity observed using the MTT assay. The damage induced at the suborganelle level could be removed by autophagy, which was maintained in the cells even after PBM. PBM at 808 nm disturbed the oxidation−reduction balance in glioblastoma cells, an effect that can be enhanced by additional stimuli, as demonstrated by our recent work combining PBM with hypericin-induced photodynamic therapy [33,58]. However, the skin response may depend on the wavelength of the irradiated light, tolerance to the light may be adjusted by various biochemical mechanisms [59], and the response to light may be altered by the frequency of treatment and by environmental triggers [60].

## 4. Conclusions

In this work, the effects of PBM on glioblastoma cells were studied because an enhancement of the photodynamic effect and a switch from autophagy to apoptosis by PBM were previously observed [33,58]. We demonstrated an increase in the concentration of proapoptotic PKCδ in the cytoplasm, as well as of cytochrome c in the mitochondria, in the PBM-treated cells. A small amount of cytochrome c was released into the cytoplasm, but apoptosis was not induced by PBM. STAT3 was not inhibited and remained in the cytoplasm of the irradiated cells. Moreover, autophagy was still active in these cells exposed to PBM. However, the extent of locally generated oxidative stress could be successfully switched in anticancer treatment through the application of additional toxins or through photodynamic therapy. Subsequently, oxidative stress could switch the autophagy signaling pathway to apoptosis.

## Data Availability

All data are presented in the text of present publication.

## References

[1] Hamblin M.R. (2018). Mechanisms and Mitochondrial Redox Signaling in Photobiomodulation. Photochem. Photobiol..

[2] Sroka R., Schaffer M., Fuchs C., Pongratz T., Schrader-Reichard U., Busch M., Schaffer P.M., Dühmke E., Baumgartner R. (1999). Effects on the mitosis of normal and tumor cells induced by light treatment of different wavelengths. Lasers Surg. Med..

[3] Murayama H., Sadakane K., Yamanoha B., Kogure S. (2012). Low-power 808-nm laser irradiation inhibits cell proliferation of a human-derived glioblastoma cell line in vitro. Lasers Med. Sci..

[4] Ravera S., Bertola N., Pasquale C., Bruno S., Benedicenti S., Ferrando S., Zekiy A., Arany P., Amaroli A. (2021). 808-nm photobiomodulation affects the viability of a head and neck squamous carcinoma cellular model, acting on energy metabolism and oxidative stress production. Biomedicines.

[5] Amaroli A., Pasquale C., Zekiy A., Utyuzh A., Benedicenti S., Signore A., Ravera S. (2021). Photobiomodulation and Oxidative Stress: 980 nm Diode Laser Light Regulates Mitochondrial Activity and Reactive Oxygen Species Production. Oxid. Med. Cell. Longev..

[6] Kausar S., Wang F., Cui H. (2018). The Role of Mitochondria in Reactive Oxygen Species Generation and Its Implications for Neurodegenerative Diseases. Cells.

[7] Murphy M.P. (2009). How mitochondria produce reactive oxygen species. Biochem. J..

[8] Chance B. (1965). Reaction of Oxygen with the Respiratory Chain in Cells and Tissues. J. Gen. Physiol..

[9] Zhao R.Z., Jiang S., Zhang L., Yu Z. (2019). Bin Mitochondrial electron transport chain, ROS generation and uncoupling (Review). Int. J. Mol. Med..

[10] Ighodaro O.M., Akinloye O.A. (2018). First line defence antioxidants-superoxide dismutase (SOD), catalase (CAT) and glutathione peroxidase (GPX): Their fundamental role in the entire antioxidant defence grid. Alex. J. Med..

[11] Zorov D.B., Juhaszova M., Sollott S.J. (2014). Mitochondrial Reactive Oxygen Species (ROS) and ROS-Induced ROS Release. Physiol. Rev..

[12] Zorov D.B., Filburn C.R., Klotz L.O., Zweier J.L., Sollott S.J. (2000). Reactive oxygen species (ROS)-induced ROS release: A new phenomenon accompanying induction of the mitochondrial permeability transition in cardiac myocytes. J. Exp. Med..

[13] Wan J., Kalpage H.A., Vaishnav A., Liu J., Lee I., Mahapatra G., Turner A.A., Zurek M.P., Ji Q., Moraes C.T. (2019). Regulation of Respiration and Apoptosis by Cytochrome c Threonine 58 Phosphorylation. Sci. Rep..

[14] Garrido C., Galluzzi L., Brunet M., Puig P.E., Didelot C., Kroemer G. (2006). Mechanisms of cytochrome c release from mitochondria. Cell Death Differ..

[15] Cereghetti G.M., Scorrano L. (2006). The many shapes of mitochondrial death. Oncogene.

[16] Martinou J.C., Youle R.J. (2011). Mitochondria in Apoptosis: Bcl-2 Family Members and Mitochondrial Dynamics. Dev. Cell.

[17] Ott M., Robertson J.D., Gogvadze V., Zhivotovsky B., Orrenius S. (2002). Cytochrome c release from mitochondria proceeds by a two-step process. Proc. Natl. Acad. Sci. USA.

[18] Zamzami N., El Hamel C., Maisse C., Brenner C., Mũoz-Pinedo C., Belzacq A.S., Costantini P., Vieira H., Loeffler M., Molle G. (2000). Bid acts on the permeability transition pore complex to induce apoptosis. Oncogene.

[19] Hayes J.D., Dinkova-Kostova A.T., Tew K.D. (2020). Oxidative Stress in Cancer. Cancer Cell.

[20] Lenkavska L., Tomkova S., Horvath D., Huntosova V. (2020). Searching for combination therapy by clustering methods: Stimulation of PKC in Golgi apparatus combined with hypericin induced PDT. Photodiagnosis Photodyn. Ther..

[21] Tomkova S., Misuth M., Lenkavska L., Miskovsky P., Huntosova V. (2018). In vitro identification of mitochondrial oxidative stress production by time-resolved fluorescence imaging of glioma cells. Biochim. Biophys. Acta—Mol. Cell Res..

[22] Misuth M., Joniova J., Horvath D., Dzurova L., Nichtova Z., Novotova M., Miskovsky P., Stroffekova K., Huntosova V. (2017). The flashlights on a distinct role of protein kinase C δ: Phosphorylation of regulatory and catalytic domain upon oxidative stress in glioma cells. Cell. Signal..

[23] Blumberg P.M. (1986). Protein Kinase C as the Receptor for the Phorbol Ester Tumor Promoters: Sixth Rhoads Memorial Award Lecture. Cancer Res..

[24] Gonzalez-Guerrico A.M., Kazanietz M.G. (2005). Phorbol ester-induced apoptosis in prostate cancer cells via autocrine activation of the extrinsic apoptotic cascade: A key role for protein kinase Cδ. J. Biol. Chem..

[25] De Vente J.E., Kukoly C.A., Bryant W.O., Posekany K.J., Chen J., Fletcher D.J., Parker P.J., Pettit G.J., Lozano G., Cook P.P. (1995). Phorbol esters induce death in MCF-7 breast cancer cells with altered expression of protein kinase C isoforms: Role for p53-independent induction of gadd45 in initiating death. J. Clin. Investig..

[26] Bond J.A., Gescher A.J., Verschoyle R.D., Lemoine N.R., Errington R., Wiltshire M., Smith P.J., Wynford-Thomas D. (2007). Cytotoxic action of phorbol esters on human pancreatic cancer cells. Int. J. Cancer.

[27] Wang Y., Biswas G., Prabu S.K., Avadhani N.G. (2006). Modulation of mitochondrial metabolic function by phorbol 12-myristate 13-acetate through increased mitochondrial translocation of protein kinase Cα in C2C12 myocytes. Biochem. Pharmacol..

[28] Huang Y.Y., Nagata K., Tedford C.E., Hamblin M.R. (2014). Low-level laser therapy (810 nm) protects primary cortical neurons against excitotoxicity in vitro. J. Biophotonics.

[29] Santulli G., Marks A. (2015). Essential Roles of Intracellular Calcium Release Channels in Muscle, Brain, Metabolism, and Aging. Curr. Mol. Pharmacol..

[30] Dikalov S.I., Li W., Doughan A.K., Blanco R.R., Zafari A.M. (2012). Mitochondrial reactive oxygen species and calcium uptake regulate activation of phagocytic NADPH oxidase. Am. J. Physiol. Integr. Comp. Physiol..

[31] Sato T., O’Rourke B., Marbán E. (1998). Modulation of mitochondrial ATP-dependent K^+^ channels by protein kinase C. Circ. Res..

[32] Majumder P.K., Pandey P., Sun X., Cheng K., Datta R., Saxena S., Kharbanda S., Kufe D. (2000). Mitochondrial translocation of protein kinase C δ in phorbol ester-induced cytochrome c release and apoptosis. J. Biol. Chem..

[33] Pevna V., Wagnières G., Huntosova V. (2021). Autophagy and apoptosis induced in u87 mg glioblastoma cells by hypericin-mediated photodynamic therapy can be photobiomodulated with 808 nm light. Biomedicines.

[34] Schneider C.A., Rasband W.S., Eliceiri K.W. (2012). NIH Image to ImageJ: 25 years of image analysis. Nat. Methods.

[35] Tatmatsu-Rocha J.C., Tim C.R., Avo L., Bernardes-Filho R., Brassolatti P., Kido H.W., Hamblin M.R., Parizotto N.A. (2018). Mitochondrial dynamics (fission and fusion) and collagen production in a rat model of diabetic wound healing treated by photobiomodulation: Comparison of 904 nm laser and 850 nm light-emitting diode (LED). J. Photochem. Photobiol. B Biol..

[36] Wang R., Dong Y., Lu Y., Zhang W., Brann D.W., Zhang Q. (2019). Photobiomodulation for Global Cerebral Ischemia: Targeting Mitochondrial Dynamics and Functions. Mol. Neurobiol..

[37] Chernivec E., Cooper J., Naylor K. (2018). Exploring the effect of rotenone—A known inducer of Parkinson’s disease—On mitochondrial dynamics in dictyostelium discoideum. Cells.

[38] Jin J., Davis J., Zhu D., Kashima D.T., Leroueil M., Pan C., Montine K.S., Zhang J. (2007). Identification of novel proteins affected by rotenone in mitochondria of dopaminergic cells. BMC Neurosci..

[39] Datta R., Heaster T.M., Sharick J.T., Gillette A.A., Skala M.C. (2020). Fluorescence lifetime imaging microscopy: Fundamentals and advances in instrumentation, analysis, and applications. J. Biomed. Opt..

[40] Silveira P.C.L., Ferreira G.K., Zaccaron R.P., Glaser V., Remor A.P., Mendes C., Pinho R.A., Latini A. (2019). Effects of photobiomodulation on mitochondria of brain, muscle, and C6 astroglioma cells. Med. Eng. Phys..

[41] Dzurová L., Petrovajova D., Nadova Z., Huntosova V., Miskovsky P., Stroffekova K. (2014). The role of anti-apoptotic protein kinase Cα in response to hypericin photodynamic therapy in U-87 MG cells. Photodiagnosis Photodyn. Ther..

[42] Misuth M., Joniova J., Belej D., Hrivnak S., Horvath D., Huntosova V. (2017). Estimation of PKCδ autophosphorylation in U87 MG glioma cells: Combination of experimental, conceptual and numerical approaches. J. Biophotonics.

[43] Singh R.K., Kumar S., Gautam P.K., Tomar M.S., Verma P.K., Singh S.P., Kumar S., Acharya A. (2017). Protein kinase C-α and the regulation of diverse cell responses. Biomol. Concepts.

[44] Kaul S., Kanthasamy A., Kitazawa M., Anantharam V., Kanthasamy A.G. (2003). Caspase-3 dependent proteolytic activation of protein kinase Cδ mediates and regulates 1-methyl-4-phenylpyridinium (MPP+)-induced apoptotic cell death in dopaminergic cells: Relevance to oxidative stress in dopaminergic degeneration. Eur. J. Neurosci..

[45] Ghayur T., Hugunin M., Talanian R.V., Ratnofsky S., Quinlan C., Emoto Y., Pandey P., Datta R., Huang Y., Kharbanda S. (1996). Proteolytic activation of protein kinase C δ by an ICE/CED 3-like protease induces characteristics of apoptosis. J. Exp. Med..

[46] Cross T., Griffiths G., Deacon E., Sallis R., Gough M., Watters D., Lord J.M. (2000). PKC-δ is an apoptotic lamin kinase. Oncogene.

[47] Larroque-Cardoso P., Swiader A., Ingueneau C., Nègre-Salvayre A., Elbaz M., Reyland M.E., Salvayre R., Vindis C. (2013). Role of protein kinase C δ in ER stress and apoptosis induced by oxidized LDL in human vascular smooth muscle cells. Cell Death Dis..

[48] Rybin V.O., Guo J., Sabri A., Elouardighi H., Schaefer E., Steinberg S.F. (2004). Stimulus-specific Differences in Protein Kinase Cδ Localization and Activation Mechanisms in Cardiomyocytes. J. Biol. Chem..

[49] Konishi H., Yamauchi E., Taniguchi H., Yamamoto T., Matsuzaki H., Takemura Y., Ohmae K., Kikkawa U., Nishizuka Y. (2001). Phosphorylation sites of protein kinase C δ in H2O2-treated cells and its activation by tyrosine kinase in vitro. Proc. Natl. Acad. Sci. USA.

[50] Kostyak J.C., Mauri B., Patel A., Dangelmaier C., Reddy H., Kunapuli S.P. (2021). Phosphorylation of protein kinase Cδ Tyr311 positively regulates thromboxane generation in platelets. J. Biol. Chem..

[51] Nakashima H., Frank G.D., Shirai H., Hinoki A., Higuchi S., Ohtsu H., Eguchi K., Sanjay A., Reyland M.E., Dempsey P.J. (2008). Novel role of protein kinase C-δ Tyr311 phosphorylation in vascular smooth muscle cell hypertrophy by angiotensin II. Hypertension.

[52] Gavish L., Gilon D., Beeri R., Zuckerman A., Nachman D., Gertz S.D. (2021). Photobiomodulation and estrogen stabilize mitochondrial membrane potential in angiotensin–II challenged porcine aortic smooth muscle cells. J. Biophotonics.

[53] Keszler A., Lindemer B., Broeckel G., Weihrauch D., Gao Y., Lohr N.L. (2022). In Vivo Characterization of a Red Light-Activated Vasodilation: A Photobiomodulation Study. Front. Physiol..

[54] Yang Q., Langston J.C., Tang Y., Kiani M.F., Kilpatrick L.E. (2019). The role of tyrosine phosphorylation of protein kinase C delta in infection and inflammation. Int. J. Mol. Sci..

[55] Kikkawa U., Matsuzaki H., Yamamoto T. (2002). Protein kinase Cδ (PKCδ): Activation mechanisms and functions. J. Biochem..

[56] Rahaman S.O., Harbor P.C., Chernova O., Barnett G.H., Vogelbaum M.A., Haque S.J. (2002). Inhibition of constitutively active Stat3 suppresses proliferation and induces apoptosis in glioblastoma multiforme cells. Oncogene.

[57] Shen S., Niso-Santano M., Adjemian S., Takehara T., Malik S.A., Minoux H., Souquere S., Mariño G., Lachkar S., Senovilla L. (2012). Cytoplasmic STAT3 Represses Autophagy by Inhibiting PKR Activity. Mol. Cell.

[58] Pevna V., Horvath D., Wagnieres G., Huntosova V. (2022). Photobiomodulation and photodynamic therapy-induced switching of autophagy and apoptosis in human dermal fibroblasts. J. Photochem. Photobiol. B Biol..

[59] Pacifico A., Damiani G., Iacovelli P., Conic R.R.Z., Scarabello A., Filoni A., Malagoli P., Bragazzi N.L., Pigatto P.D.M., Morrone A. (2020). Photoadaptation to ultraviolet B TL01 in psoriatic patients. J. Eur. Acad. Dermatol. Venereol..

[60] Pacifico A., Conic R.R.Z., Cristaudo A., Garbarino S., Ardigò M., Morrone A., Iacovelli P., Di Gregorio S., Pigatto P.D.M., Grada A. (2021). Diet-related phototoxic reactions in psoriatic patients undergoing phototherapy: Results from a multicenter prospective study. Nutrients.

